# Stress-related genes promote *Edwardsiella ictaluri* pathogenesis

**DOI:** 10.1371/journal.pone.0194669

**Published:** 2018-03-19

**Authors:** Ali Akgul, Ayfer Akgul, Mark L. Lawrence, Attila Karsi

**Affiliations:** 1 Department of Basic Sciences, College of Veterinary Medicine, Mississippi State University, Mississippi State, MS, United States of America; 2 Department of Clinical Sciences, College of Veterinary Medicine, Mississippi State University, Mississippi State, MS, United States of America; Instituto Butantan, BRAZIL

## Abstract

*Edwardsiella ictaluri* is a Gram-negative facultative anaerobic rod and the causative agent of enteric septicemia of channel catfish (ESC), which is one of the most prevalent diseases of catfish, causing significant economic losses in the catfish industry. *E*. *ictaluri* is resistant to complement system and macrophage killing, which results in rapid systemic septicemia. However, mechanisms of *E*. *ictaluri* stress responses under conditions of host environment are not studied well. Therefore, in this work, we report *E*. *ictaluri* stress responses during hydrogen peroxide, low pH, and catfish serum stresses as well as during catfish invasion. *E*. *ictaluri* stress responses were characterized by identifying expression of 13 universal stress protein (USP) genes (*usp01*-*usp13*) and seven USP-interacting protein genes (*groEL*, *groES*, *dnaK*, *grpE*, and *clpB*, *grpE*, *relA*). Data indicated that three *usp* genes (*usp05*, *usp07*, and *usp13*) were highly expressed in all stress conditions. Similarly, *E*. *ictaluri* heat shock proteins *groEL*, *groES*, *dnaK*, *grpE*, and *clpB* were highly expressed in oxidative stress. Also, *E*. *ictaluri grpE* and *relA* were highly expressed in catfish spleen and head kidney. These findings contribute to our understanding of stress response mechanisms in *E*. *ictaluri* stress response, and stress-related proteins that are essential for *E*. *ictaluri* could be potential targets for live attenuated vaccine development against ESC.

## Introduction

*E*. *ictaluri* is a Gram-negative bacterium causing enteric septicemia of channel catfish (ESC), one of the most important diseases of farm-raised channel catfish in the US [1, 2]. *E*. *ictaluri* is a facultative intracellular pathogen capable of surviving inside catfish macrophages [3], which contributes rapid spread of the pathogen [4]. *E*. *ictaluri* virulence factors include flagella [5], extracellular capsular polysaccharide [6], lipopolysaccharide [7–11] outer membrane proteins [11–15], hemolysins [12] and chondroitinase [13]. Recently, it was shown that *E*. *ictaluri* lipopolysaccharide oligo-polysaccharide (LPS O-PS) plays a major role during catfish infection [14], and *E*. *ictaluri* tricarboxylic acid (TCA) cycle and ferric hydroxamate uptake systems also contribute to *E*. *ictaluri* virulence in catfish [15–18].

Bacteria often have to cope with stressful conditions such as nutrient limitation, abiotic stresses, and predation. Bacterial adaptation to stress includes regulation of mRNA stability and translation, protein quality control systems, and specific and general stress responses. Understanding bacterial stress responses contribute to our knowledge of host microenvironments, mechanisms of virulence, and potential targets for treatment of infectious diseases [19].

Universal stress proteins (USPs) are a conserved group of stress proteins that are generally between 140–160 amino acids. They are present in archaea, bacteria, plants, and fungi, but not humans [20]. *E*. *coli* has six *usp* genes whose products are involved in several functions including oxidative stress, adhesion, and motility [21]. Expression of *usp* genes is regulated by sigma factors within RNA polymerases, and ppGpp is another important regulator of USPs [22]. Under stress, USPs are upregulated and aid bacteria in surviving stressful conditions [20, 23].

USPs are important for pathogenic bacteria. For example, *Salmonella*, *Klebsiella*, and *Mycobacterium* need USPs to invade the host [24]. USPs are also involved in persistence and intracellular survival of *Mycobacterium tuberculosis* [25]; growth arrest and virulence in *Salmonella* [26]; virulence of *Burkholderia pseudomallei* [27]; and intracellular adaptation of *Listeria monocytogenes* [28]. In *Staphylococcus aureus*, virulence factors were downregulated in vivo while expression of *uspA* increased [29]. In *Acinetobacter baumannii*, *uspA* is essential for pneumonia and sepsis pathogenesis [30].

USPs can interact with several heat shock proteins (HSPs) and other stress-related proteins to help bacterial virulence and stress response. Therefore, we determined the expression of 13 *usp* (*usp01* to *usp13*) and seven USP-interacting genes, including six genes that encode heat shock proteins (*groEL*, *groES*, *dnaK*, *dnaJ*, *clpB*, and *grpE*) and a gene that encodes guanosine pentaphosphate synthetase (*relA*). We expect that expression analysis of these genes will help us understand stress responses in *E*. *ictaluri*, and stress-related proteins that are essential for *E*. *ictaluri* could be potential targets for live attenuated vaccine development against ESC.

## Materials and methods

### Ethics statement

Animal work was performed in accordance with the Institutional Animal Care and Use Committee at Mississippi State University. The Office of Laboratory Animal Resources at Mississippi State University is required to adhere to applicable federal, state, local and institutional laws and policies governing animal research.

### Bacteria, plasmids, and growth conditions

Bacteria and plasmids used in this study are shown in Table 1. *E*. *ictaluri* strain 93–146 was grown at 30°C using brain heart infusion (BHI) broth and agar (Difco, Sparks, MD). *E*. *coli* were cultured at 37°C using Luria-Bertani (LB) broth and agar (Difco). *E*. *coli* SM10λ*pir* were used for transferring pAK*gfplux*1 into *E*. *ictaluri*. Ampicillin (100 μg/ml) and colistin (12.5 μg/ml) antibiotics (Sigma- Aldrich, Saint Louis, MN) were used when needed.

**Table 1:** Bacterial strains and plasmids used.

Strain	Relevant characteristics	References
*Edwardsiella ictaluri* 93–146	Wild type; pEI1+; pEI2+; Col^r^	[31]
*Escherichia coli* SM10λ*pir*	thi; thr; leu; tonA; lacY; supE; recA;::RP4-2-Tc::Mu; Kmr; lpirR6K	[32]
**Plasmids**		
pAK*gfplux*1	11547 bp, pBBR1MCS4, *gfpmut3*, *luxCDABE*	[33]

### Identification of stress-related proteins

Sequences of the *E*. *ictaluri* USP genes and proteins were obtained from the *E*. *ictaluri* genome [34], and six heat shock proteins (GroL, GroS, DnaJ, DnaK, ClpB, and GrpE) interacting with USPs were identified using STRING v10 [35]. The STRING database (http://string-db.org) provides pre-calculated protein-protein interactions for organisms, including *E*. *ictaluri*. By querying protein sequences of the *E*. *ictaluri* USPs, the interacting proteins were identified, and the interaction network was visualized. Because of its regulatory role for some USPs, ppGpp synthetase (relA) was also included in our analysis.

### Acid stress (low pH exposure)

An *E*. *ictaluri* strain 93–146 colony was inoculated in 5 ml of BHI broth, followed by 16–18 h incubation at 30^°^C with shaking. The next day, 40 ml of BHI broth was inoculated and grown to an optical density (OD_600_) of 0.4. Then each culture was divided into four aliquots, 10 ml each, and bacteria were harvested by centrifugation at 6,000 x g for 15 m. Supernatant was removed, and bacteria were resuspended in acidic BHI broth (pH 4.0, acidified with 6 N HCl solution) and standard BHI broth (pH 7.0). Cultures were incubated with shaking (180 rpm) at 30^°^C for 30 m., and pellets were collected by centrifugation (7000 x g for 15 m). RNAlater was added, and bacteria were stored for a week at -20^°^C until RNA was isolated.

### Oxidative stress

*E*. *ictaluri* 93–146 was streaked on BHI agar containing 12.5 μg/ml of colistin and incubated at 30^°^C for 48 h. Small cultures were prepared by inoculating 5 ml of BHI broth with 12.5 μg/ml colistin and incubating for 16-18h at 30^°^C with shaking at 200 rpm. 40 ml of BHI broth were inoculated and grown to an optical density (OD600) of 0.4. Each culture was divided into four aliquots, 10 ml each, and bacteria were harvested by centrifugation at 6,000 x g for 15 m. Supernatant was removed, and bacteria were resuspended in two types of BHI broth: 20 ml standard BHI broth and 20 ml BHI broth supplemented with 1.5 mM (0.05%) H_2_O_2_. Cultures were incubated with shaking (180 rpm) at 30^°^C for 30 m. Bacteria were harvested by centrifugation at 7000 x g for 15 m., and pellets were suspended in RNAlater and stored a week at -20^°^C until RNA isolation.

### Serum stress

*E*. *ictaluri* were exposed to naïve catfish serum and heat-inactivated catfish serum. Each treatment included four biological replicates. *E*. *ictaluri* cultures were washed three times using 1.25 ml of cell wash buffer (10 mM TrisCl and 5 mM magnesium acetate). Either normal serum or heat-inactivated serum (1.25 ml) was added to each *E*. *ictaluri* pellet. Tubes were inverted to mix bacteria and serum thoroughly followed by incubation for 30 m at 30^°^C. Serum-bacteria mixture was used for total RNA isolation.

### In vivo gene expression

Approximately 18-month-old SPF channel catfish (19.7 cm, 18.5 g) were stocked into two tanks at a rate of 7 fish/tank. After one week of acclimation, fish were anesthetized in water containing 100 mg/L tricaine methanesulfonate (MS-222), and bioluminescent *E*. *ictaluri* were injected into intraperitoneal space in 100 μl PBS (approximately 1×10^4^ CFU). Negative control fish were injected with 100 μl PBS.

Bacteria were visualized in live catfish using an IVIS Lumina XRMS In Vivo Imaging System (PerkinElmer, Waltham, MA) by following the procedures described previously [36]. Bioluminescence imaging (BLI) was conducted at 3, 6, 12, 24 and 30 h post-infection. At each time point, catfish were anesthetized in water containing 100 mg/L MS222 and transferred immediately to the photon collection chamber. Total photon emissions from the whole fish body were collected at an exposure time of 30 s. Following BLI imaging, fish were returned to well-aerated water for recovery. Bioluminescence was quantified using Living Image Software v 4.2 (Caliper Life Sciences., Hopkinton, Massachusetts), and mean photon intensity for each treatment was used in statistical analysis.

At 30 h post-exposure, fish were euthanized with a high dose of MS-222 (300 mg/L), and anterior kidney and spleen tissues were collected immediately in RNAlater Stabilization Solution. Then, RNA was isolated from four fish with an RNeasy Mini Kit (Qiagen).

### Total RNA isolation

In each experiment, total RNA was isolated from four biological replicates using RNeasy Protect Bacteria Mini Kit (Qiagen). Contaminating bacterial DNA was eliminated by DNase I treatment with RNase-Free DNase Set (Qiagen). The concentration and quality of the isolated total RNA were measured by NanoDrop 1000 (Thermo Scientific). First-strand cDNA was produced from 1 μg total RNA using Maxima First Strand cDNA Synthesis Kit for RT-qPCR (Thermo Scientific) by following manufacturer’s instructions.

### Real-time qPCR

Primers sequences and expected PCR fragment sizes are shown in Table 2. Optimal annealing temperatures of primers were determined by gradient PCR. Primer specificities were determined by melting curve analysis and visual inspection of PCR products on agarose gel. After assessment of primers’ quality, quantitative real-time PCR (qRT-PCR) was performed using a Mx3005P qPCR System (Agilent Technologies, CA, USA) and DyNAmo SYBR Green qPCR Kit (Finnzymes Oy, Espoo, Finland). Each 20 μl PCR reaction contained 10 μl SYBR Green 2X mix, 0.2 μM each of forward and reverse primers, and one μl of 100 x diluted cDNA. The PCR was set to initial denaturation at 95°C for 3 m, 45 cycles of denaturation at 95°C for 15 s, annealing at 60°C for 30 s, and extension at 72°C for 30 s, and a final extension at 72°C for 3 m. At the end of the PCR, a melting curve program from 60°C to 95°C with 0.5°C increase every 15 s was run. 16S RNA gene was used as internal control. Bacteria grown in BHI broth were used as experimental control while control group in serum experiment was bacteria exposed to heat-treated serum.

**Table 2:** Primers used for expression analysis.

Gene Name	Gene ID	Primer	Size	Sequence (5`-3`)	Product (bp)	Locus ID
*usp01*	*-*	F	21	TCTACAGGCGTTGGCTGATAC	126	NT01EI_1627
		R	21	ATCAGGATCAGGTCGGCATC		
*usp02*	*uspF*	F	20	TGAACACACCCATTTCGAGA	175	NT01EI_1751
		R	18	CCGGTCTATGGCGCAAAG		
*usp03*	*-*	F	21	AGGACGCTCATATCCATTTCC	190	NT01EI_1786
		R	21	GAGCCGATGGCAATAGTACAG		
*usp04*	*uspE*	F	21	GATATTCTGGCAGCAGAGTGC	134	NT01EI_1962
		R	21	TCTGCAACCTCTTTCAACACC		
*usp05*	*uspA*	F	20	GAAAATTGACGGTGATGGCG	203	NT01EI_1981
		R	20	GCTGAGCGTCAATCCATCAA		
*usp06*	*uspA*	F	22	CTACCACGATCCACTGAACATC	165	NT01EI_2616
		R	22	CGAATAGCCTCGTTGTAGATCC		
*usp07*	*kdpD*	F	21	CGCATGTCCAGAAATTACCAT	147	NT01EI_2891
		R	21	GCCACGGAAAGAAATGAGATA		
*usp08*	*uspA*	F	22	ATGTTCTGGTTCTGATTCATGG	150	NT01EI_3729
		R	22	TCAGATTGACATCCAGCATACC		
*usp09*	*uspA*	F	21	CTGGTGATGGTCTACCTGCTC	186	NT01EI_3778
		R	21	AATACCGACGATCAGCATCAC		
*usp10*	*-*	F	20	AGAATTCCAGGTGTAGCGGT	110	NT01EI_1634
		R	19	GCAGTAACGGCGCTCATAG		
*usp11*	*-*	F	21	GTTAATCTGGGCGATATGCAA	195	NT01EI_1803
		R	21	GCTCCAGAAGTCCTGATGATG		
*usp12*	*uspB*	F	20	TACTGACACAGAATGCGTTGG	120	NT01EI_3728
		R	20	TACCAAATGAGTAGGCGGTGA		
*usp13*	*cpxP*	F	20	AGAGTTTGATCCTGGCTCAG	147	NT01EI_3810
		R	19	GGTTACCTTGTTACGACTT		
*clpB*	*clpB*	F	21	CCAAGTATTTACCGTCCGCAA	166	NT01EI_3205
		R	21	ACCCTGAGCTGTTTTAATGCG		
*dnaJ*	*dnaJ*	F	20	CTGAGTGCGATGTCTGCCAC	123	NT01EI_0673
		R	20	AAGGCTGCTGTACGGTGAAG		
*dnaK*	*dnaK*	F	21	TACAACATGGATCTGACGCTG	186	NT01EI_0672
		R	20	CTGTACGGTGAAGAAGCCCT		
*GroEL*	*groL*	F	20	GTGGTATCGTGAAAGTGGCG	124	NT01EI_0380
		R	21	AGCTCCATACCGATCTCTTCA		
*groES*	*groS*	F	20	TTGAGTCCAAGTCTGCTGGC	162	NT01EI_0379
		R	21	ACCATCGTTGAAAATCACGGT		
*grpE*	*grpE*	F	21	GATGTCGAGAAGGCGCACAAG	158	NT01EI_3062
		R	21	AGCAGGGATTTCAGGGTCAG		
*relA*	*ppGpp synthetase*	F	20	CGGGTGAAGGATTACATCGC	170	NT01EI_0032
		R	21	GACTCGCCCTCTTTATACGCC		
*16sRNA*	*-*	F	20	AGAGTTTGATCCTGGCTCAG	150	NT01EI_0010
		R	19	GGTTACCTTGTTACGACTT		

### Statistical analysis

Relative expression values were calculated by the threshold cycle (Ct) changes in sample and control using the ΔΔCt method [37]. All expression values were normalized against 16S rRNA. Relative expression was determined by the comparative Ct method of relative quantification (RQ), which was calculated with the formula 2^-ΔΔCt^. ΔΔCt was calculated by ΔΔCt = ΔCt (stress condition) - ΔCt (non-stress condition), where ΔCt is the normalized signal level in a sample (ΔCt = Ct of target gene–Ct of reference gene).

One-way Analysis of Variance (ANOVA) was used to compare gene expression among different stress conditions with SAS 9.1.4 (SAS Institute, Cary, NC). P value cutoff for statistical significance was < 0.05. To increase statistical reliability, four replicates were run for each sample, and the mean Ct considered for each gene.

## Results

### *E*. *ictaluri* USPs and stress-related proteins

**Fig 1:**
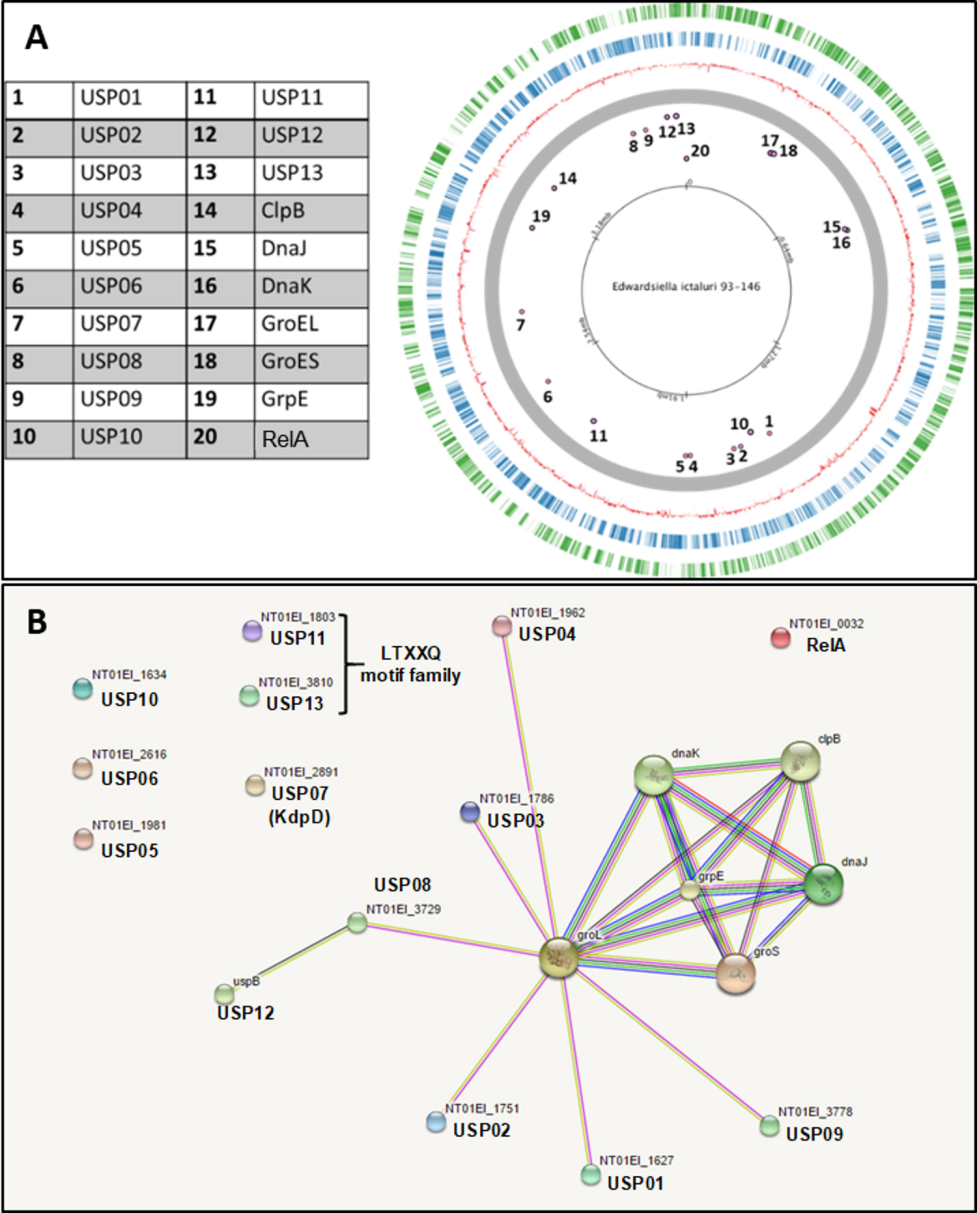
Locations of *E*. *ictaluri* stress-related genes and their protein network. (A) Locations of studied genes in *E*. *ictaluri* genome. (B) Functional protein association network showing the relationship between USPs and heat shock proteins in *E*. *ictaluri*.

We identified 13 USPs and seven stress-related proteins encoded in the *E*. *ictaluri* genome [34] (Fig 1A). Six heat shock proteins (GroL, GroS, DnaJ, DnaK, ClpB, and GrpE) interacting with USPs were also identified by using STRING10 [35] (Fig 1B). ppGpp synthetase (RelA) was also included because of its regulatory role for some USPs.

### Bioluminescence imaging (BLI) of live catfish

**Fig 2:**
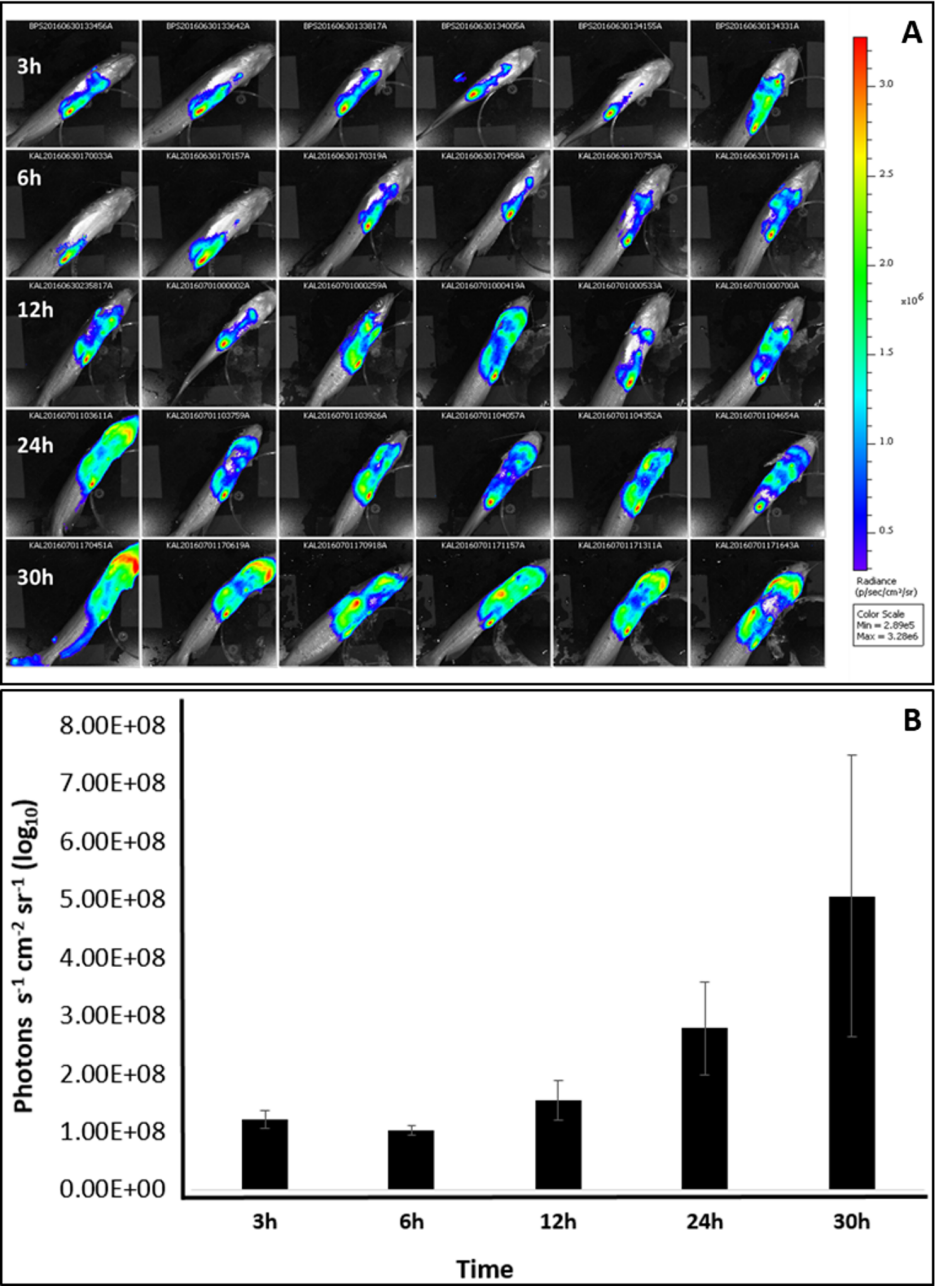
Bioluminescence imaging of live catfish. (A) Catfish injected with bioluminescent *E*. *ictaluri*. Times of imaging are shown on the left, and a scale bar showing photon intensities is on the right. (B) Mean photon emissions from six catfish at each time point.

Imaging of bioluminescent *E*. *ictaluri* in live catfish allowed us to determine the best time point for tissue collection (Fig 2A and 2B). After a brief decline at 6 h post-injection, average photon intensity, and hence bacteria number, increased continuously, which was approximately 5-fold higher at 30 h compared to initial imaging at 3 h post-injection. BLI showed clearly that *E*. *ictaluri* colonized most internal organs of catfish at 30 h post-injection, and it was an appropriate time for collection of anterior kidney and spleen tissues.

### Expression analysis of stress-related genes in acid stress

**Fig 3:**
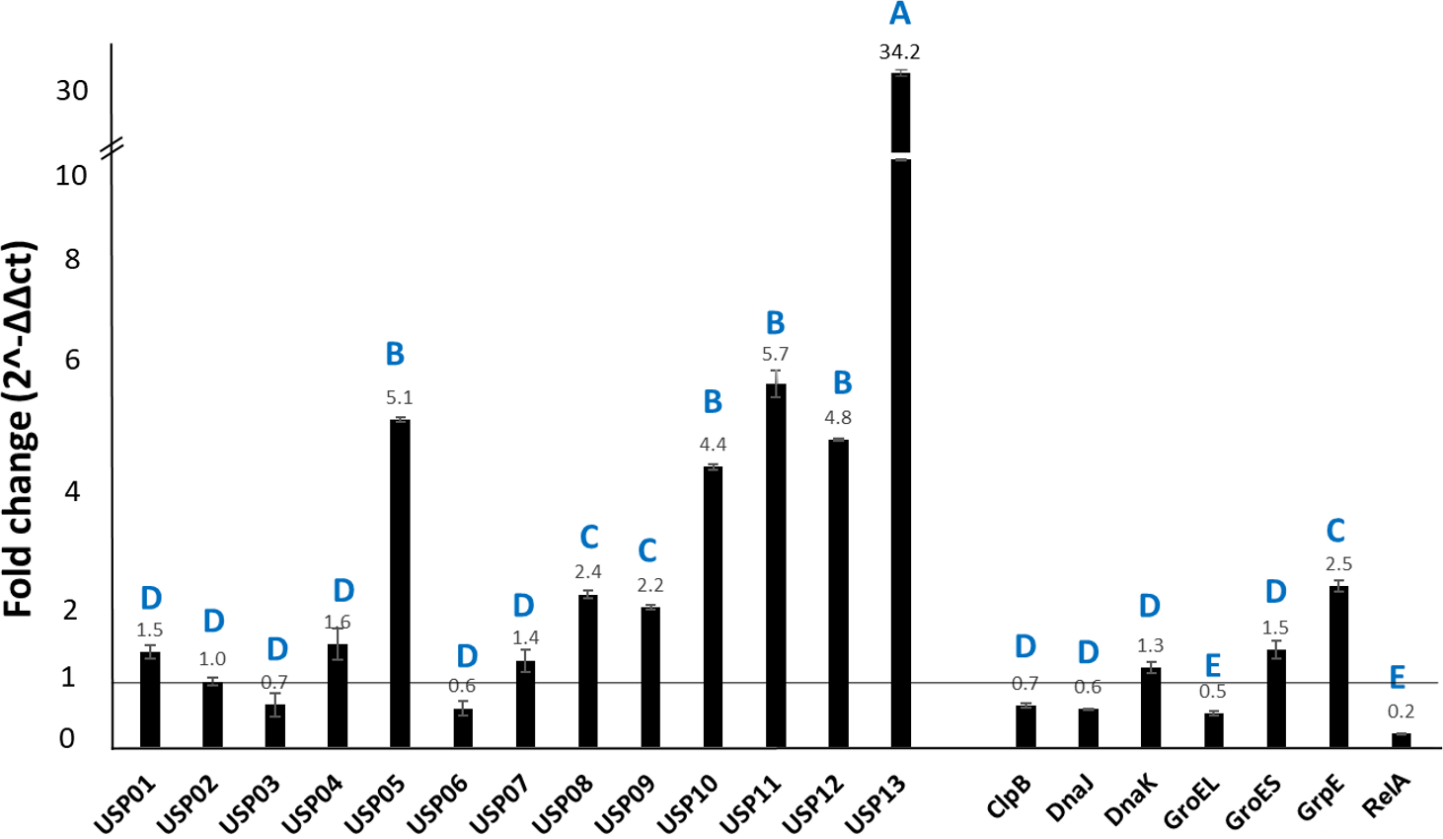
Relative expression of stress-related genes in *E*. *ictaluri* exposed to low pH stress. Bars above the vertical line at fold change 1 indicate upregulation and bars below the vertical line at fold change 1 indicate downregulation. Letters indicate statistical groupings at significance level *P* < 0.05.

At pH of 4.0, *usp13* showed the highest expression level with 34-fold increase relative to non-stress condition. Additionally, *usp05* and *usp11* expressions increased more than 5-fold while the increase was more than 2-fold in *usp08*, *usp09*, *usp10*, *usp12*, and *grpE* (Fig 3).

### Expression analysis of stress-related genes in oxidative stress

**Fig 4:**
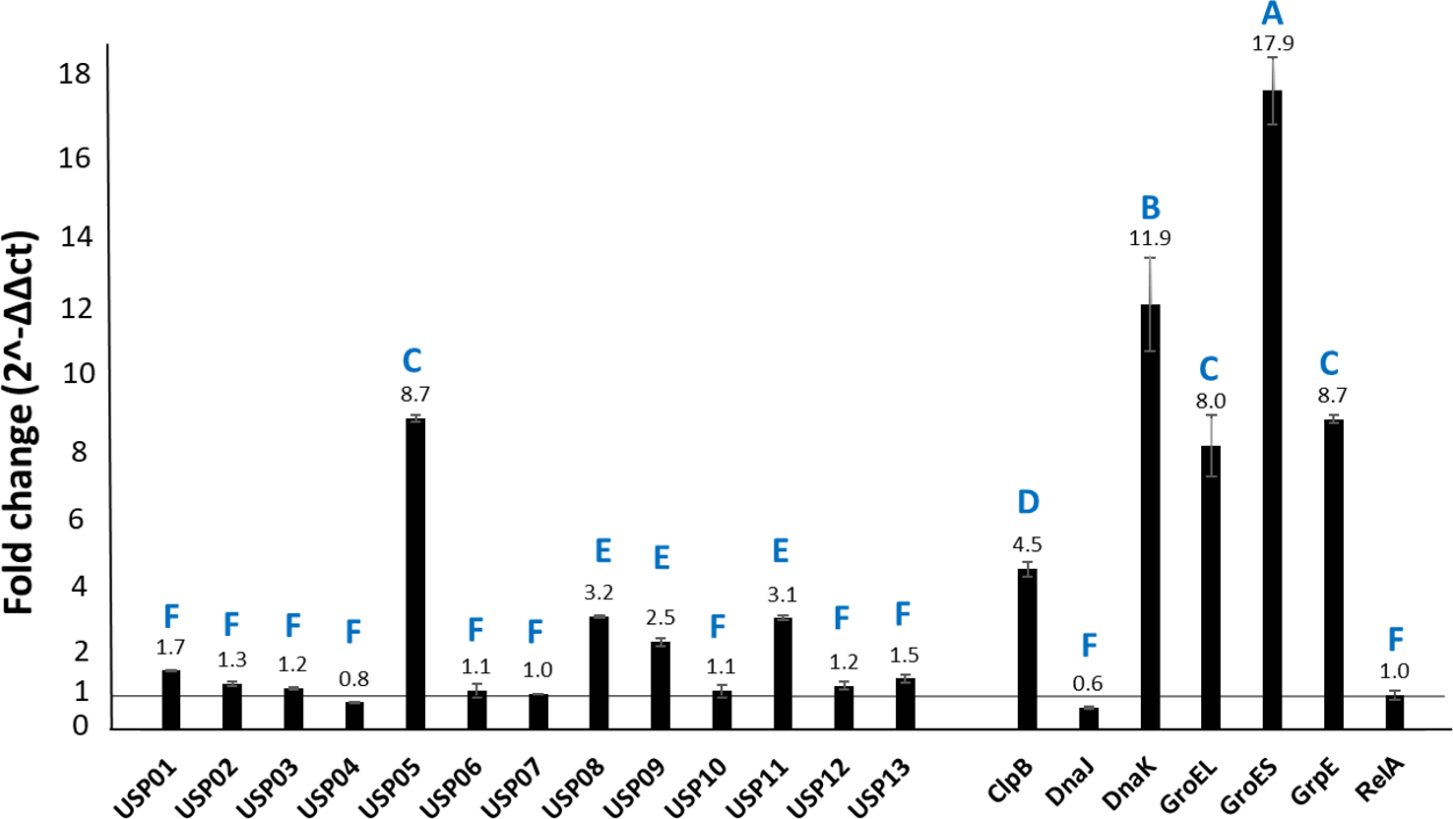
Relative expression of stress-related genes in *E*. *ictaluri* exposed to H_2_O_2_. Bars above the vertical line at fold change 1 indicate upregulation and bars below the vertical line at fold change 1 indicate downregulation. Letters indicate statistical groupings at significance level *P* < 0.05.

In 0.05% H_2_O_2_, *groES* and *dnaK* expression increases were 18- and 11-fold, respectively, which was significantly greater than other genes. *usp05*, *groEL*, and *grpE* showed more than 8-fold; *clpB* showed 4.5 fold; and *usp08*, *usp09*, and *usp11* exhibited more than 2-fold increased expression (Fig 4).

### Expression analysis of stress-related genes in serum

**Fig 5:**
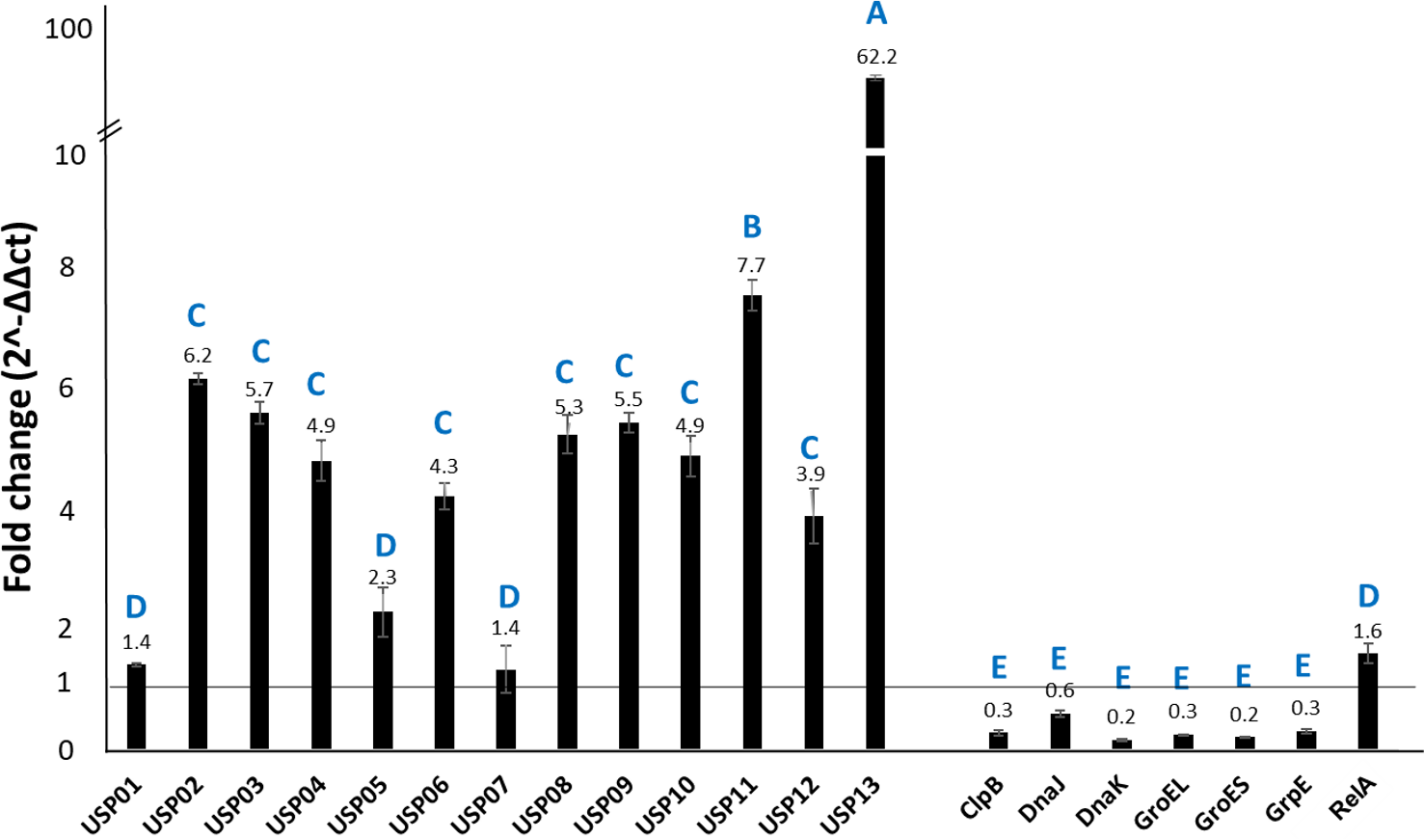
Relative expression of stress-related genes in *E*. *ictaluri* exposed to naïve catfish serum and heat-treated serum. Bars above the vertical line at fold change 1 indicate upregulation and bars below the vertical line at fold change 1 indicate downregulation. Letters indicate statistical groupings at significance level *P* < 0.05.

In normal catfish serum, *usp13* showed the highest expression level with 62.2-fold compared to heat-inactivated serum. The *usp11* had 7.7-fold; *usp02*, *usp03*, *usp04*, *usp06*, *usp08*, *usp09*, *usp10*, and *usp12* had more than 4-fold; and *usp05* showed more than 2-fold increased expression (Fig 5).

### Expression analysis of stress-related genes in catfish

**Fig 6:**
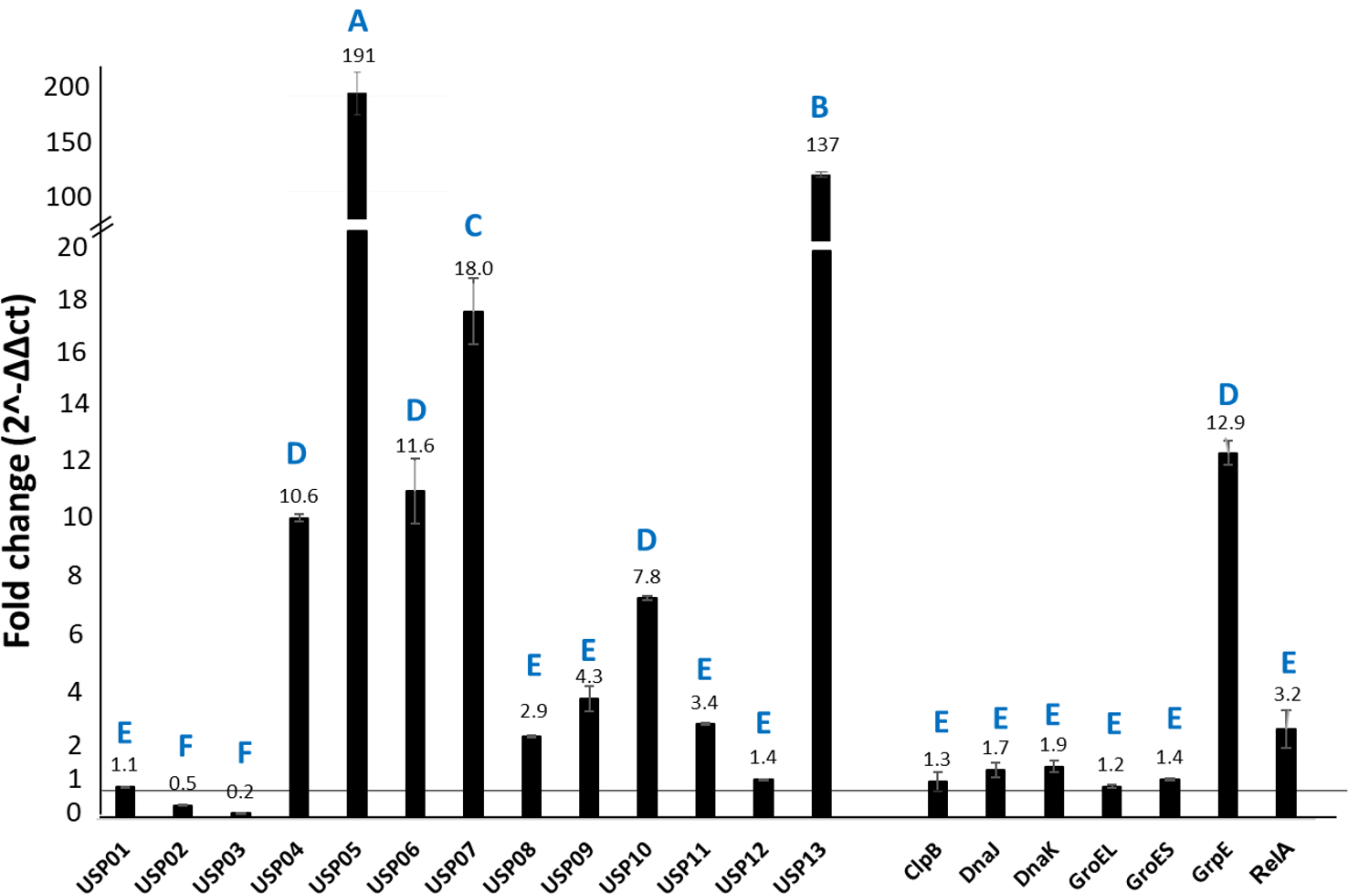
Relative expression of *E*. *ictaluri* stress-related genes in catfish spleen. Bars above the vertical line at fold change 1 indicate upregulation and bars below the vertical line at fold change 1 indicate downregulation. Letters indicate statistical groupings at significance level *P* < 0.05.

**Fig 7:**
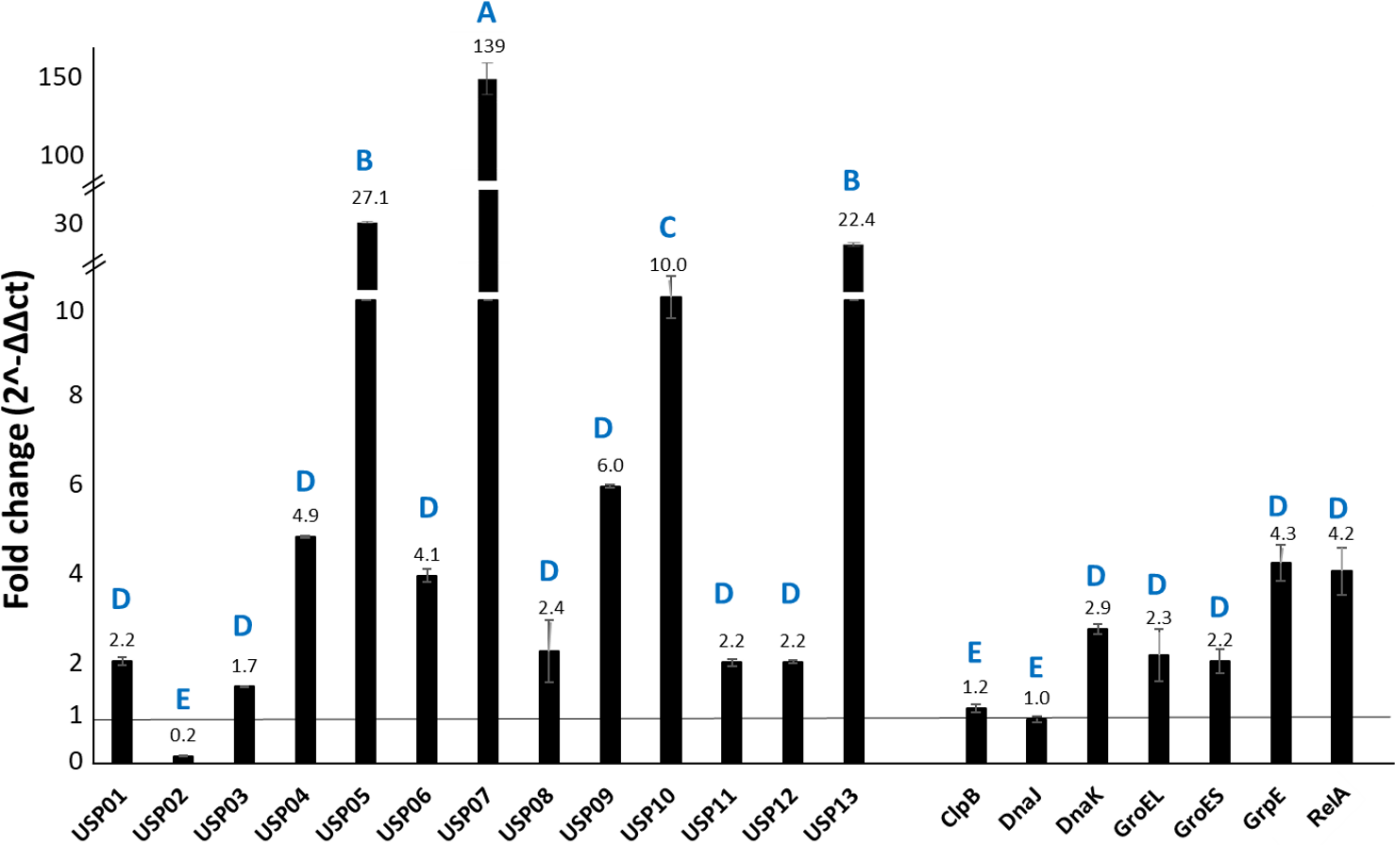
Relative expression *E*. *ictaluri* stress-related genes in infected catfish kidney. Bars above the vertical line at fold change 1 indicate upregulation and bars below the vertical line at fold change 1 indicate downregulation. Letters indicate statistical groupings at significance level *P* < 0.05.

**Fig 8:**
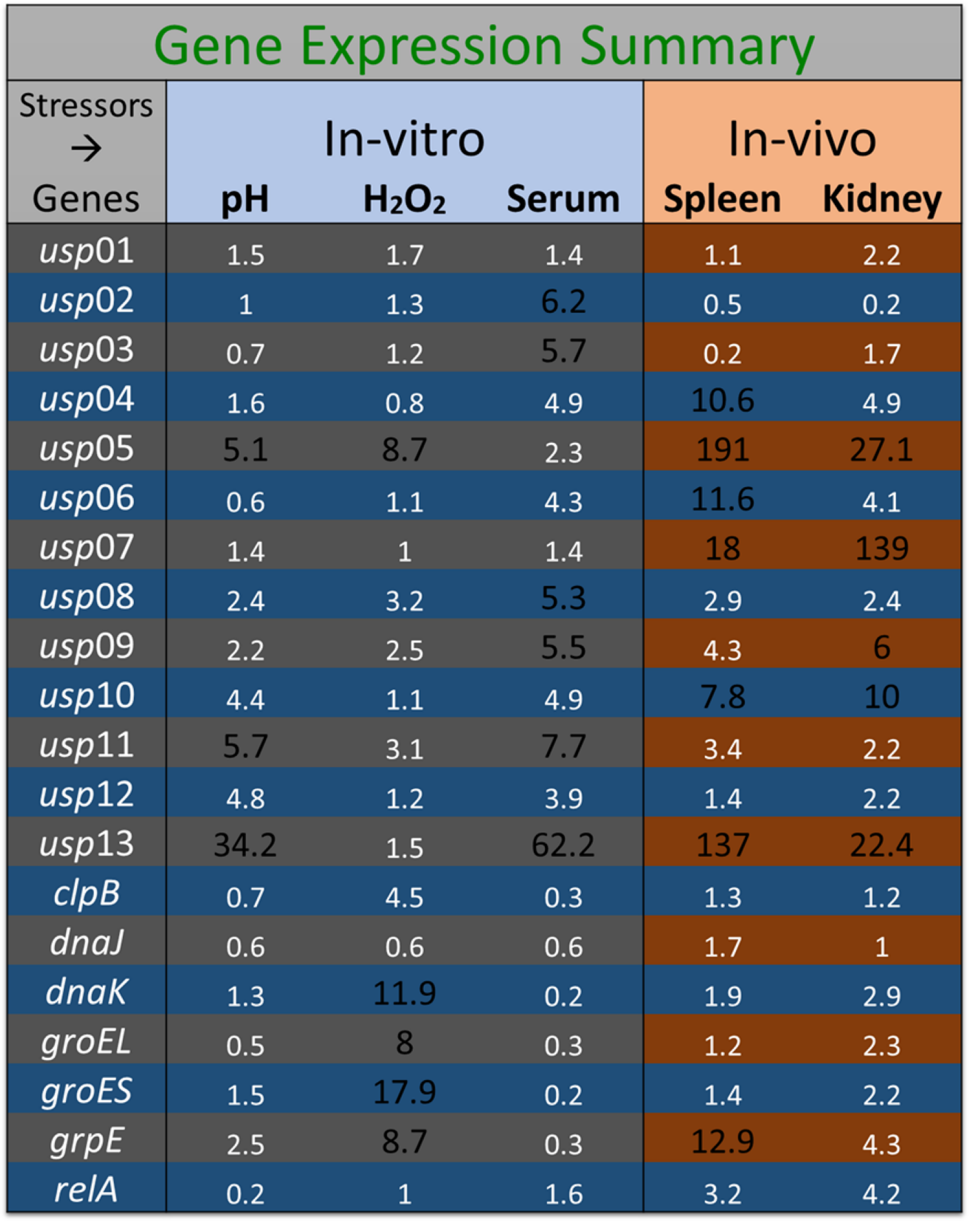
Summary of relative expression of *E*. *ictaluri* stress genes in various stress conditions. Relative expression values above 5-fold were shown in a larger and bold font.

Expression of the 20 stress response genes was determined in catfish spleen and head kidney at 30 h post-exposure relative to *E*. *ictaluri* grown in BHI broth. The *usp05* and *usp13* genes showed the highest expression levels in the spleen with 191- and 137- fold increase, respectively. Expression increases of other genes in the spleen were: *usp07* 18-fold; *usp04*, *usp06*, *usp10*, and *grpE* more than 7.8 fold; and *usp08*, *usp09*, *usp11*, and *relA* more than 2-fold (Fig 6). In head kidney, *usp07* showed the highest expression level with 139-fold increase relative to control (Fig 7). Increased expressions of other genes were *usp05* and *usp13* more than 22-fold; *usp10* more 10-fold; *usp04*, *usp06*, *usp09*, *grpE*, and *relA* more than 4-fold; and *usp08*, *usp11*, *usp12*, *dnaK*, *groEL*, and *groES* more than 2 fold. A summary of all experiments was provided in Fig 8.

## Discussion

In this research, we aimed to determine the expression of 20 stress-related genes under different stress conditions. There are different types of universal stress proteins (USP) in archaea, bacteria, and plants [22]. *E*. *coli* has six USP groups (USPA, USPB, USPC, USPD, USPE, and USPF), which are involved in several functions including oxidative stress, adhesion, and motility [21].

Following bacterial phagocytosis by macrophages, fusion of the lysosome with the phagosome causes release of acid-activated enzymes and toxic metabolites and acidification of the phagolysosome to pH 4–5. As a result, bacteria are typically inactivated and broken down by the enzymes and toxins. *E*. *ictaluri* can resist macrophage killing and replicate in phagocytes, which is considered one of the key mechanisms for rapid spread of the pathogen in the host. The low pH and oxidative stress we applied mimic the conditions present in the phagolysosome [38]. Expression increase in *usp05*, *usp13*, *dnaK*, *groEL*, *groES*, and *grpE* may indicate their involvement in *E*. *ictaluri* survival in harsh phagolysosomal conditions.

Expression of *usp* genes in serum was upregulated, while expression of HSPs was mostly down-regulated. In particular, *usp13* expression was 62.2-fold compared to control. The complement system in serum lyses and opsonizes bacteria for phagocytosis. The complement system of fish can also inactivate bacterial exotoxins [39]. In fish, three pathways of complement activation have been identified: the classical complement pathway (CCP), alternative complement pathway (ACP), and the lectin complement pathway (LCP) [40]. *E*. *ictaluri* is resistant to serum [41]. In *E*. *ictaluri*, sialic acid-induced suppression of ACP activation was thought to be the mechanism for *E*. *ictaluri* resistance to normal catfish serum [41]. Besides, a larger amount of surface proteins and polysaccharide material were present compared to virulent strains [6]. These surface components help prevent complement and lysozyme in normal serum, from contacting vulnerable sites on the bacterial surface [39]. Because of *E*. *ictaluri*’s ability to survive in serum, we were not expecting a high expression of stress response genes in normal serum. Only *usp13* showed significant up-regulation, suggesting that USP13 might be associated with serum tolerance in *E*. *ictaluri*.

Imaging of live catfish injected with bioluminescent *E*. *ictaluri* indicated a similar infection pattern shown in our previous studies [17, 36]. Bioluminescence imaging shows a brief decline of photon intensity at early hours of infection, which is probably due to strong immune responses of catfish against *E*. *ictaluri*. Later, photon intensity increases faster due to rapid colonization of catfish by the pathogen. This rapid increase may indicate overwhelming of catfish immune system by the pathogen, which results in death of catfish in a couple of days. Therefore, 30 h post-infection seems a suitable time point for tissue collection because *E*. *ictaluri* is in a rapid increase stage while catfish immune responses are not overwhelmed completely.

Whereas *usp05*, *usp08*, *usp09*, and *usp11* were highly expressed under in vitro stress conditions, *usp04-08*, *usp10*, *usp13*, *grpE*, and *relA* were upregulated in the host. Overall, *usp05*, *usp07*, *usp13*, and *grpE* may be critical for *E*. *ictaluri* to cope with several different stressors. Previously, our group identified a transposon insertion mutant of *usp05* that showed attenuated virulence in catfish and provided protection against ESC. The *usp05* gene is known as *uspA*, and it is an important regulator of survival and virulence in many pathogens [23]. An *E*. *coli uspA* mutant caused a survival defect under a variety of growth-arrested conditions, whereas overexpression induces growth in the growth-arrested state. Our data suggest that *usp05* could be an important virulence gene in *E*. *ictaluri*.

Kdp is a K^+^ transporter system in *E*. *coli*, and KdpD/E control the expression of the *kdpABC* operon, which is one of two-component sensor-regulator [42]. USP07 is a KdpD protein, and it contains a USPA domain [43]. We included the whole KdpD as a USP07 because USP domain is located between the N terminal sensor domain and C-terminal catalytic domain of this Osmosensitive K+ channel histidine kinase. Biochemical studies revealed that USPC interacts specifically with the USP domain in the stimulus perceiving N-terminal domain of KdpD, and UspC stabilized the KdpD/KdpE, and DNA complex to act as a scaffolding protein under salt stress in *Staphylococcus aureus* [44]. KdpD functions as a membrane-associated protein kinase that phosphorylates KdpE in response to environmental signals [43]. *Salmonella enterica* serovar Typhimurium *kdpD* mutant is attenuated in host and macrophage survival experiment. Also, it is promoting resistance to osmotic, oxidative, and antimicrobial stresses [45]. KdpD is involved oxidative-osmotic stress response to host and bacterial virulence [46]. In our study, *usp07* showed very high expression, especially when *E*. *ictaluri* is in the catfish head kidney and spleen, indicating that it may involve in *E*. *ictaluri* survival in the host.

USP13 was described as a universal stress protein and CpxP like protein in NCBI. USP13, extracytoplasmic adaptor protein (CpxP), is placed in the inner membrane with histidine kinase CpxA and CpxR, a response regulator [47, 48]. CpxP is a highly inducible member of the Cpx regulon, and its expression was elevated in responses to envelope stress and entry into stationary phase growth [49, 50]. Also, CpxP functions as an adaptor protein, carrying misfolded periplasmic proteins to the DegP protease for degradation [51, 52]. CpxP is degraded along with its misfolded substrate, which suggests that bacteria can posttranslationally modulate CpxP levels [47]. Cpx system is important and required for virulence in both Gram-negative and -positive bacteria [53]. CpxP also modulates the activity of the Cpx system by dynamic interaction with CpxA in response to specific stresses [54]. On the other hand, CpxP overexpression results in a reduced Cpx-response [55], hence interfering with the induction of envelope stress response. Thereby, CpxP inhibits autophosphorylation of reconstituted CpxA [56]. According to the current model, the inhibitory and supporting functions of CpxP for envelope stress response are linked. In unstressed cells, CpxP associates with CpxA to shut off the Cpx-TCS. Envelope-stress conditions induce the displacement of CpxP from CpxA resulting in Cpx-TCS activation [54]. In *E*. *ictaluri*, CpxP showed very high expression in all stress conditions except oxidative stress. CpxP is an important regulator of cell membrane stress in bacteria during host infection, and it might promote the virulence and survival mechanisms related to distinct salt and acidic conditions.

Other seven stress proteins were chosen by a possible regulation of USPs or interaction with USPs in *E*. *ictaluri*. Protein-protein interactions play critical roles in protein function, and in a large scale protein-protein interaction study in *E*. *coli* showed that a USPA has 83 target proteins, which mostly overlap with DnaK, DnaJ, and GroEL, also interactions with GroEL and DnaK (HSP70) [57]. GroEL and DnaK may require both systems for folding, degradation, or translocation [57]. The function of GroEL/ES is in protein folding and possibly in intercellular signaling, and prevention of misfolding under stress conditions was determined [58]. DnaK/J plays an essential role in DNA replication and participates actively in response to hyperosmotic shock. DnaK is also involved in refolding heat-damaged proteins [19]. GroEL was previously determined with various other proteins as a related protein to the bacterial virulence in *E*. *tarda* [59]. In another proteomics study comparing two strains of *E*. *tarda* with different degrees of virulence, authors showed that GroEL was highly expressed in pathogenic strain compared to nonpathogenic strain [60]. Our result indicated that expression of *groEL and groES* were high in the oxidative stress, and *dnaK* was highly upregulated. Therefore, highly expressed heat shock proteins in oxidative stress should further investigated to explain the folding and unfolding process in oxidative stress of *E*. *ictaluri*. ClpB plays an important role in protein control to protein disaggregation in collaboration with the DnaK, DnaJ, and GrpE [50], and it is sensitive to heat shock and other extreme stresses [61, 62]. In *E*. *tarda*, protein expression of ClpB was higher in virulent strain than non-virulent [60]. In our study, the expression of *clpB* was high in oxidative stress like *dnaK*, *groEL*, and *groES*. ClpB is part of a stress-induced system involved in the recovery of the cell from damage in cooperation with DnaJ, DnaK, and GrpE. The *grpE* was also shown similar high expression in oxidative stress and the internal organs. Further, it has very high expression in low pH. Thus, *grpE* may be an important heat shock protein to cope with stress in *E*. *ictaluri*.

The stringent response (SR) coordinates adaptations to nutritional starvation and various stress conditions [63, 64]. SR relies on the ‘alarmones’ ppGpp, and pppGpp synthetases [65]. The ppGpp acts by modifying the activity of many cellular targets including DNA replication, transcription, translation, ribosome assembly and metabolism [63, 65, 66]. Also, it is recently found that it has a role in DNA repair mechanism [67]. The SR is in the center bacterial survival and virulence [66, 68, 69]. In our study, we determined that the expression of ppGpp synthetase (*relA*) was increased when *E*. *ictaluri* was in kidney and spleen. Similarly, serum affects the expression of *relA*. Therefore, we think that *relA* could be essential for *E*. *ictaluri* adaptation in the host. Further studies should be conducted in *relA* to better understand its role in *E*. *ictaluri* virulence and the regulation of USPs.

In this study, we identified that three *usp* genes (*usp 05-07-13*) were highly expressed in many stress conditions, and five heat shock proteins were highly expressed in oxidative stress (*groEL/ES*, *dnaK*, *grpE* and *clpB*), and *grpE* was highly expressed in spleen, kidney, and low pH. Finally, *relA* was highly expressed in kidney and spleen. As a result, we expect these findings will help us understand the role of stress proteins in *E*. *ictaluri* virulence. Furthermore, essential USPs and stress regulators in *E*. *ictaluri* can be potential targets for live vaccine development against ESC.
